# 

**Published:** 1917-12-01

**Authors:** 


### CONTENTS.

PAGE

**The Royal Statistical Society's National Value** 173

**Prompt Action by Voluntary Hospitals Essential** ... ... ... ... ... ... ... 174

**Hospital and Institutional News** ... ... ... 175
Doctors as Politicians—The Children's Hospital To-day—The Treloar Children's Hospital—Maternity Home for Unmarried Women—Medical Students in the French Army—Fatigue and the Worker—The Grouping of District Councils—Orthopædic Centres for Discharged Soldiers—The Man who "Does Nothing"—Soap for Wounds—The Cumulative Endowment of Beds—Wanted: German Science?—Chestnuts for Munitions—Death of a Well-known Hospital Officer—The Royal Gwent Hospital—The Gas-driven Motor-car—A New Method of Anæsthesia—The Impossible—This Week's Drug Market.

**The Prophylaxis of Venereal Diseases**... ... 179
An Offence Against the General Interest.

**The Story of the Red Cross** ... ... ... 181

By the Hon. Sir Arthur Stanley, G.B.E., M.P., Chairman J.W.C.

**National Insurance Act Developments**... ... 183
The Amendment Bill Now Before Parliament.

**The Institutional Library** ... ... ... ... ... 185

A Masterly Hospital History.

PAGE

**The Editor's Letter-Box** ... ... ... ... 187
Westminster Hospital's Special Appeal—"Is Thy Secretary a Dog?"—A Pharmacist's Contribution to Hospital Economy.

**Nursing Affairs in Ireland** ... ... ... ... 188

**Parliament and the Profession** ... ... ... 188

**The Matrons' and Sisters' Department** ... 189
The College of Nursing and its Opponents: A Remarkable Volte Face.
Round the Hospitals.
The College Going Ahead—Its Opponents Confronted with Facts—The Humbug of Agitators' Methods—Enthusiastic Meetings at Leicester and Manchester—The Mechanic in the Kitchen—A Home Sister of the Bygones.

**Some Hospital Types of British Nursing** ... 191
II. A Home Sister of the Bygones.

**Bringing Down the Infantile Death-Rate** ... 193
The Priestley Lecture of the National Health Society.

**Matrons' and Sisters' Appointments** ... ... 194

**Passing Events and Topics** ... ... ... ... 194

**The Institutional Worker** ... .. (Suppl., 1

Starting an Auxiliary Military Hospital.

Question for December.

Questions Invited.

